# Pioglitazone Protects Mesenchymal Stem Cells against *P*-Cresol-Induced Mitochondrial Dysfunction via Up-Regulation of PINK-1

**DOI:** 10.3390/ijms19102898

**Published:** 2018-09-24

**Authors:** Yeo Min Yoon, Yong-Seok Han, Chul Won Yun, Jun Hee Lee, Rang Kim, Sang Hun Lee

**Affiliations:** 1 Medical Science Research Institute, Soonchunhyang University Seoul Hospital, Seoul 336-745, Korea; yoonboo15@naver.com (Y.M.Y); format7000@naver.com (Y.-S.H); skydbs113@naver.com (C.W.Y); 2 Department of Pharmacology and Toxicology, University of Alabama at Birmingham School of Medicine, Birmingham, AL 35294, USA; j-school@hanmail.net; 3 Department of Human Development, Cornell University College of Human Ecology, Ithaca, NY 14850, USA; rk522@cornell.edu; 4 Departments of Biochemistry, Soonchunhyang University College of Medicine, Cheonan 330-930, Korea

**Keywords:** mesenchymal stem cell, pioglitazone, PTEN-induced putative kinase 1, cell proliferation, chronic kidney disease

## Abstract

Mesenchymal stem cells (MSC) could be a candidate for cell-based therapy in chronic kidney disease (CKD); however, the uremic toxin in patients with CKD restricts the therapeutic efficacy of MSCs. To address this problem, we explored the effect of pioglitazone as a measure against exposure to the uremic toxin *P*-cresol (PC) in MSCs. Under PC exposure conditions, apoptosis of MSCs was induced, as well as PC-induced dysfunction of mitochondria by augmentation of mitofusion, reduction of mitophagy, and inactivation of mitochondrial complexes I and IV. Treatment of MSCs with pioglitazone significantly inhibited PC-induced apoptosis. Pioglitazone also prevented PC-induced mitofusion and increased mitophagy against PC exposure through up-regulation of phosphatase and tensin homolog (PTEN)-induced putative kinase 1 (PINK-1). Furthermore, pioglitazone protected against PC-induced mitochondrial dysfunction by increasing the cytochrome c oxidase subunit 4 (COX4) level and activating complexes I and IV, resulting in enhancement of proliferation. In particular, activation of nuclear factor κ-light-chain-enhancer of activated B cells (NF-κB) regulated the pioglitazone-mediated up-regulation of PINK-1. These results indicate that pioglitazone protects MSCs against PC-induced accumulated mitochondrial dysfunction via the NF-κB–PINK-1 axis under *P*-cresol exposure conditions. Our study suggests that pioglitazone-treated MSCs could be a candidate for MSC-based therapy in patients with CKD.

## 1. Introduction

Mesenchymal stem cells (MSCs) are a promising source of stem-cell-based therapeutics for regenerative medicine because they have the potential for self-renewal and multipotent differentiation [1]. MSC transplantation has shown therapeutic efficacy in several disease models, such as myocardial infarction, hind limb ischemia, wound healing, and stroke, owing to their potential for expansion, differentiation, and secretion of cytokines [2]. Although MSCs have a high therapeutic potential for regenerative diseases, their transplantation and therapeutic efficacy are limited by the pathophysiological conditions in damaged sites. Glucose deprivation, oxidative stress, ischemia, and inflammation lead to dysfunction and affect the survival of transplanted MSCs, resulting in the cell death of transplanted cells in the targeted area [3,4]. Therefore, to promote the therapeutic effects of MSCs in various diseases, it is important to improve the survival of engrafted MSCs in damaged sites by endowing them with resistance to these pathophysiological conditions.

Chronic kidney disease (CKD) is the progressive loss of kidney function. CKD leads to dysfunction of organs and systems, including the cardiovascular system, central nervous system, immune system, endocrine system, gastro-intestinal system, and respiratory system, as well as hematological and coagulation disorders and bone disease [5]. In particular, damaged kidneys secrete uremic toxins, resulting in complications from the accumulation of toxic products [6]. The uremic toxin *P*-cresol (PC) is a low-molecular-weight uremic compound with high affinity to protein; it originates from tyrosine and phenylalanine catabolism by intestinal microorganisms [7]. Recent studies have shown that PC reduces the functionalities of MSCs [8,9]. PC induces cell apoptosis by PC-mediated oxidative stress [10]. Furthermore, PC suppresses mitochondrial respiration, leading to cellular damage in CKD patients [11,12]. Therefore, it is important to enhance MSC functionality against uremic toxins for successful MSC-based therapy in CKD patients.

Pioglitazone, which is commonly used in type 2 diabetes and works by reducing insulin resistance, is an agonist of peroxisome proliferator-activated receptor (PPAR)-γ [13]. Recent study has suggested that pioglitazone reduces inflammation and oxidative stress in neuron cells through improvements in mitochondrial function [14]. However, it is unknown whether pioglitazone has a protective effect on MSCs against PC through the protection of mitochondrial function. To investigate the effect of pioglitazone on MSCs against the uremic toxin PC, we confirmed the effect of PC on MSCs.

## 2. Results

### 2.1. Protective Effect of Pioglitazone against PC-Induced Apoptosis in MSCs

**Figure 1:**
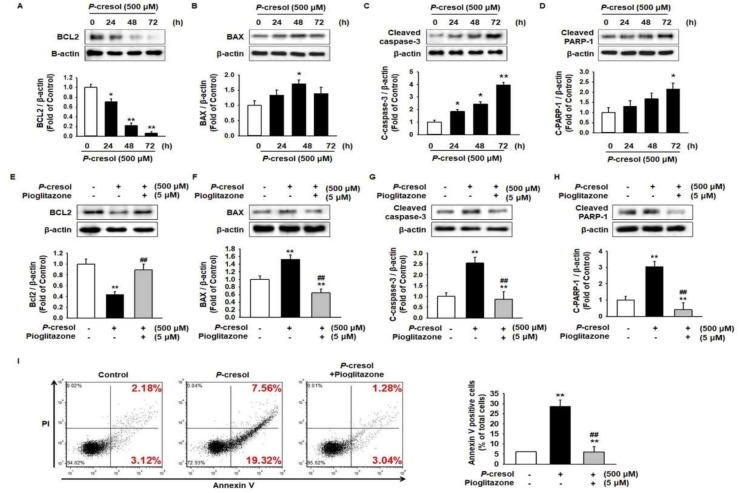
Pioglitazone-treated mesenchymal stem cells (MSCs) and induced apoptosis on exposure to *P*-cresol (PC). (**A**–**D**) Western blot was used to analyze the expression of apoptosis-associated proteins BCL2, BAX, cleaved caspase-3, and cleaved PARP-1 in MSCs exposed to PC (500 μM) for 0, 24, 48, and 72 h. The expression levels were determined by densitometry relative to β-actin. Values represent the mean ± standard error of the mean (SEM). * *p* < 0.05, and ** *p* < 0.01 vs untreated MSCs. (**E**–**H**) Western blot was used to analyze MSCs pretreated with pioglitazone (5 μM; 24 h) for the expression of apoptosis-associated proteins BCL2, BAX, cleaved caspase-3, and cleaved PARP-1 on exposure to PC. The expression levels were determined by densitometry relative to β-actin. Values represent the mean ± SEM. ** *p* < 0.01 vs untreated MSCs, ## *p* < 0.01 vs MSCs exposed to PC. (**I**) Annexin V and PI positive cells were detected using fluorescence-activated cell sorting (FACS) analysis. Values represent the mean ± SEM. ** *p* < 0.01 vs untreated MSCs, ## *p* < 0.01 vs MSCs exposed to PC.

To determine the apoptosis pathway in MSCs under PC exposure conditions, the levels of apoptosis-associated proteins, including B-cell lymphoma 2 (BCL2), Bcl-2-associated X protein (BAX), cleaved capsase-3, and cleaved Poly(ADP-ribose) polymerase 1 (PARP-1), were analyzed after treatment of MSCs with PC for 0, 24, 48, and 72 h. The expression of the anti-apoptosis protein BCL2 was significantly decreased and that of the pro-apoptosis proteins BAX, cleaved caspase-3, and cleaved PARP-1 significantly increased after treatment with PC in a time-dependent manner (Figure 1A–D). To investigate the effect of pioglitazone on MSC characterization, we analyzed MSC surface markers such as cluster differentiation 29 (CD29), CD90, CD105 (MSC-specific markers as positive markers), and CD34 (hematopoietic marker as a negative marker) post treatment of MSCs with pioglitazone. There was no significant change in the expressions of surface markers between control and pioglitazone-treated MSCs (Supplemental Figure S1). To verify the protective effect of pioglitazone on apoptosis in MSCs against PC, the expressions of apoptosis proteins were assessed after pretreatment of MSCs with pioglitazone. Pioglitazone protected against the PC-induced reduction of BCL2 and the PC-induced augmentation of BAX, cleaved caspase-3, and cleaved PARP-1 levels (Figure 1E–H). In addition, Annexin V/PI staining showed that pioglitazone inhibited PC-induced apoptosis (Figure 1I).

### 2.2. Pioglitazone Protects against PC-Induced Reduction of the PINK-1 Level through Regulation of NF-κB

**Figure 2:**
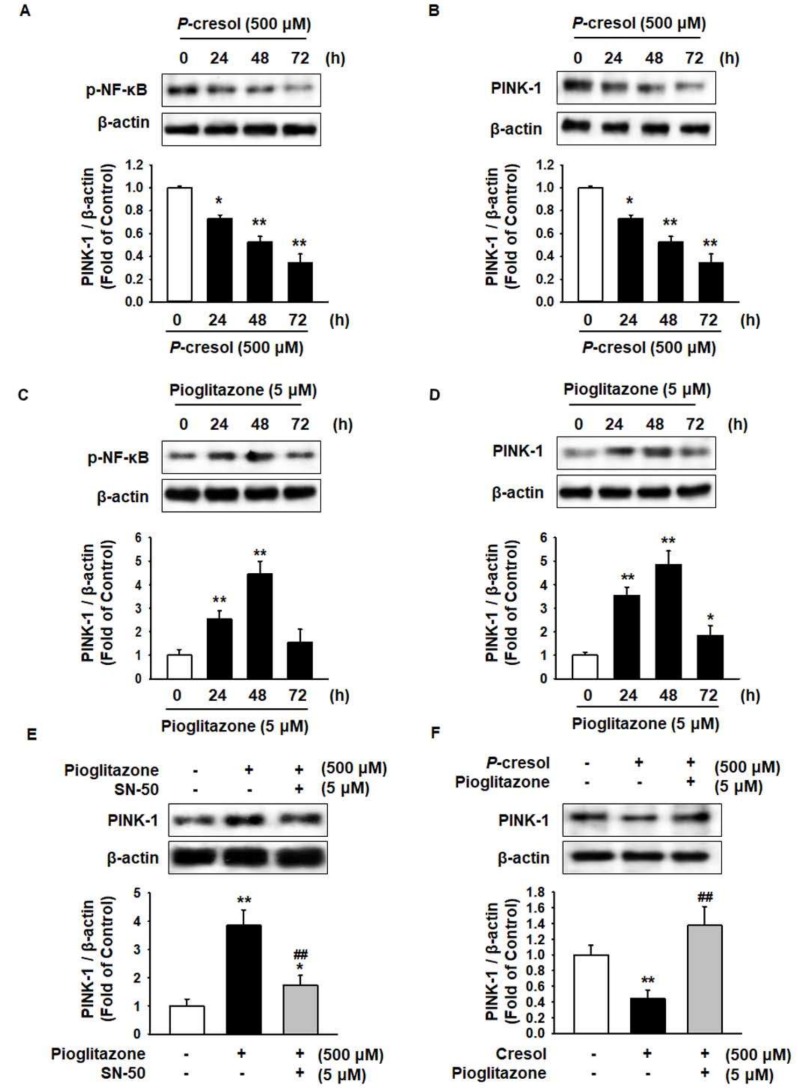
Pioglitazone-treated MSCs increased the expression of PINK-1 through activation of NF-κB. (**A**,**B**) Western blot was used to analyze the activation of p-NF-κB and the expression of PINK-1 in MSCs on exposure to PC (500 μM) for 0, 24, 48, and 72 h. The expression levels were determined by densitometry relative to β-actin. Values represent the mean ± SEM. * *p* < 0.05 and ** *p* < 0.01 vs untreated MSCs. (**C**,**D**) Western blot was used to analyze the activation of p-NF-κB and the expression of PINK-1 in MSCs treated with pioglitazone (5 μM) for 0, 24, 48, and 72 h. The expression levels were determined by densitometry relative to β-actin. Values represent the mean ± SEM. * *p* < 0.05 and ** *p* < 0.01 vs untreated MSCs. (**E**) Pretreated MSCs with or without NF-κB SN50 cell permeable inhibitory peptide trifluoroacetate salt (SN-50) for 24 h were analyzed via Western blot for the expression of PINK-1 in the treatment with pioglitazone (5 μM; 48 h). The expression levels were determined by densitometry relative to β-actin. Values represent the mean ± SEM. * *p* < 0.05 and ** *p* < 0.01 vs untreated MSCs, ## *p* < 0.01 vs MSCs treated with pioglitazone. (**F**) MSCs pretreated with or without pioglitazone (5 μM; 48 h) were analyzed via Western blot for the expression of PINK-1 on exposure to PC (500 μM; 48 h). The expression levels were determined by densitometry relative to β-actin. Values represent the mean ± SEM. ** *p* < 0.01 vs untreated MSCs, ## *p* < 0.01 vs MSCs exposed to PC.

Phosphatase and tensin homolog-induced kinase 1 (PINK-1) selectively eliminates mitochondrial dysfunction by mitophagy and regulates expression by nuclear factor κ-light-chain-enhancer of activated B cells (NF-κB) [15]. To investigate the effect of PC on PINK-1 in MSCs, the levels of p-NF-κB and PINK-1 were analyzed. The phosphorylation of NF-κB and the expression of PINK-1 significantly decreased after treatment of MSCs with PC in a time-dependent manner (Figure 2A,B). To explore the effect of pioglitazone on NF-κB and PINK-1 in MSCs, the expression of p-NF-κB and PINK-1 was measured after treatment with pioglitazone for 0, 24, 48, and 72 h. The expressions of p-NF-κB and PINK-1 significantly increased after treatment with pioglitazone for 48 h (Figure 2C,D). To investigate whether NF-κB regulates PINK-1 after treatment with pioglitazone, we analyzed the expression of PINK-1 after pretreatment with NF-κB SN50 cell permeable inhibitory peptide trifluoroacetate salt (SN-50), which is an NF-κB inhibitor. This resulted in an increase in the PINK-1 level via activation of NF-κB (Figure 2E). Furthermore, under PC exposure conditions, pioglitazone ameliorated the PC-induced reduction of PINK-1 (Figure 2F).

### 2.3. Pioglitazone Maintains Mitochondrial Dynamics through Expression of PINK-1

**Figure 3:**
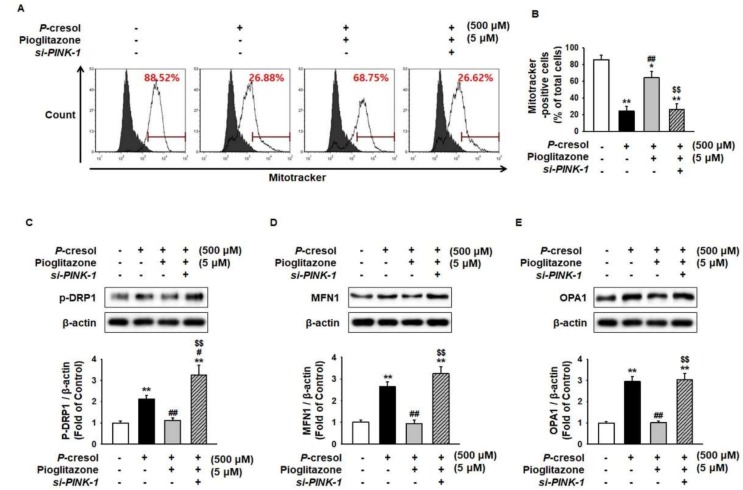
Pioglitazone-treated MSCs increased mitochondrial fission by increasing expression of PINK-1 on exposure to PC. (**A**,**B**) After PC (500 μM) exposure for 72 h, mitochondria were measured using Mitotracker in *si-PRNP*-transfected MSCs after treatment with pioglitazone (5 μM) for 24 h. Values represent the mean ± SEM. * *p* < 0.05 and ** *p* < 0.01 vs untreated MSCs, ## *p* < 0.01 vs MSCs exposed to PC, $$ *p* < 0.01 vs MSCs treated with pioglitazone. (**C**–**E**) After the 72 h PC (500 μM) exposure, Western blot was used to analyze the activation of phosphorylated dynamin-related protein 1 (p-DRP1) and the expression of mitofusin-1 (MFN1) and dynamin-like 120 kDa protein (OPA1) in MSCs after 24 h of treatment of small interfering-RNA PRioN Protein (*si-PRNP*)-transfected MSCs with pioglitazone (5 μM; 24 h). The expression levels were determined by densitometry relative to β-actin. Values represent the mean ± SEM. ** *p* < 0.01 vs untreated MSCs, # *p* < 0.05 and ## *p* < 0.01 vs MSCs exposed to PC, $$ *p* < 0.01 vs MSCs treated with pioglitazone.

In CKD patients, mitochondrial size is increased by disruption, mitochondrial fission/fusion dynamics, and limited autophagosome elimination [16]. Thus, enhanced expression of mitofusion-associated proteins indicates harmful effects in CKD [17]. Mitotracker analysis has shown that PC significantly decreased Mitotracker-positive cells (Figure 3A,B), suggesting that the amount of mitochondria decreased owing to mitofusion. To investigate whether PC induces mitofusion, the regulation of mitofusion-associated proteins, including phosphorylated dynamin-related protein 1 (p-DRP1), mitofusin-1 (MFN1), and dynamin-like 120 kDa protein (OPA1), was assessed. The activation of mitofusion-associated proteins was significantly increased under the PC exposure condition (Figure 3C–E). Under PC exposure conditions, pioglitazone blocked mitofusion through regulation of PINK-1 (Figure 3A–E).

**Figure 4:**
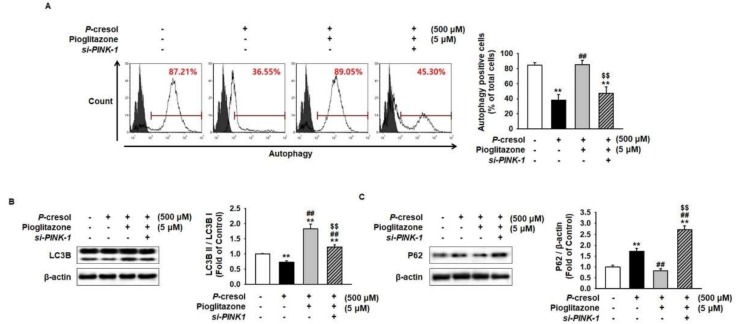
Pioglitazone-treated MSCs increased mitophagy by increasing PINK-1 on exposure to PC. (**A**) Autophagy was measured by lysosomal/autophagic vacuole fluorescent staining. Values represent the mean ± SEM. ** *p* < 0.01 vs untreated MSCs, ## *p* < 0.01 vs MSCs exposed to PC, $$ *p* < 0.01 vs MSCs treated with pioglitazone. (**B**,**C**) After the 72 h PC (500 μM) exposure, Western blot was used to analyze the expression of LC3B and P62 in MSCs after 24 h treatment of *si-PRNP*-transfected MSCs with pioglitazone (5 μM; 24 h). The expression levels were determined by densitometry relative to β-actin. Values represent the mean ± SEM. ** *p* < 0.01 vs untreated MSCs, ## *p* < 0.01 vs MSCs exposed to PC, $$ *p* < 0.01 vs MSCs treated with pioglitazone.

To further explore whether pioglitazone regulates the autophagy process in MSCs under PC exposure conditions, we performed autophagy assay and analyzed the expression of microtubule-associated proteins 1A/1B light chain 3B (LC3B) and nucleoporin 62 (P62) after treatment of MSCs with pioglitazone. Autophagy assay has shown that PC significantly inhibited autophagy with respect to the control (Figure 4A). In addition, the expression of LC3BII significantly decreased and the P62 level significantly increased in PC-treated MSCs, compared with the control (Figure 4B,C). However, pioglitazone counteracted PC-mediated inhibition of autophagy via PINK-1 expression (Figure 4A–C).

### 2.4. Pioglitazone Enhances MSC Proliferation through the Electronic Transport Chain

**Figure 5:**
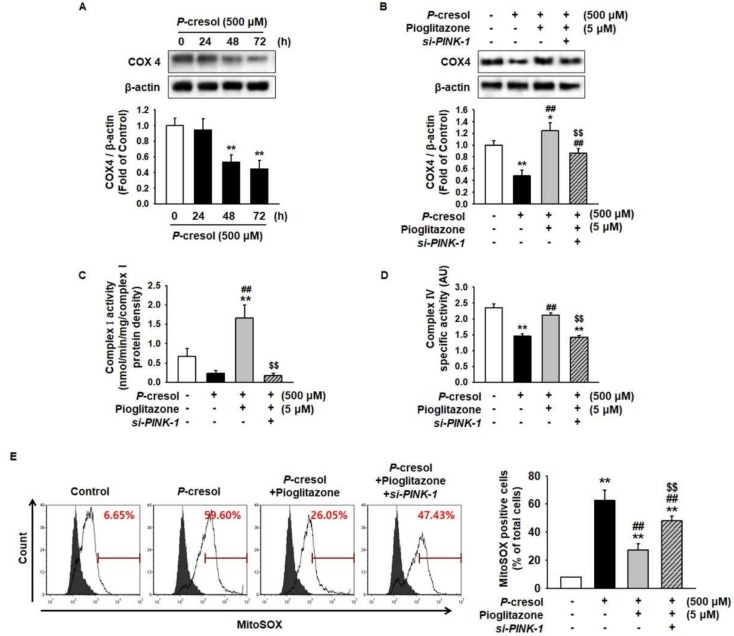
Pioglitazone-treated MSCs restored mitochondrial function by increasing PINK-1. (**A**) Western blot was used to analyze the expression of cytochrome c oxidase subunit 4 (COX4) in MSCs on exposure to PC (500 μM) for 0, 24, 48, and 72 h. The expression levels were determined by densitometry relative to β-actin. Values represent the mean ± SEM. ** *p* < 0.01 vs untreated MSCs. (**B**) After the 72 h PC (500 μM) exposure, Western blot was used to analyze the expression of COX4 in MSCs after the 24 h treatment in *si-PRNP*-transfected MSCs with pioglitazone (5 μM; 24 h). The expression levels were determined by densitometry relative to β-actin. Values represent the mean ± SEM. * *p* < 0.05 and ** *p* < 0.01 vs untreated MSCs, ## *p* < 0.01 vs MSCs exposed to PC, $$ *p* < 0.01 vs MSCs treated with pioglitazone. (**C**,**D**) After exposure to PC (500 μM; 72 h), complex I and IV activity was measured in the MSCs treated with pioglitazone (5 μM; 24 h) by evaluating the expression of PINK-1. Values represent the mean ± SEM. ** *p* < 0.01 vs untreated MSCs, ## *p* < 0.01 vs MSCs exposed to PC, $$ *p* < 0.01 vs MSCs treated with pioglitazone. (**E**) Mitochondrial ROS was detected by MitoSOX™. The positive MitoSOX™ of MSCs was measured using a flow cytometer. Values represent the mean ± SEM. ** *p* < 0.01 vs untreated MSCs, ## *p* < 0.01 vs MSCs exposed to PC, $$ *p* < 0.01 vs MSCs treated with pioglitazone.

Previous study has shown that PINK-1 regulated the expression of cytochrome c oxidase subunit 4 (COX4) and activation of electronic transport chain complex I and IV activity [18]. To establish whether pioglitazone is involved in the activity of the electronic transport chain in MSCs under the PC exposure condition, we analyzed the level of COX4 and the activity of mitochondrial complexes I and IV. PC significantly inhibited the expression of COX4 in MSCs in a time-dependent manner (Figure 5A,B). In addition, the activity of mitochondrial complexes I and IV was inhibited by treatment with PC (Figure 5C,D). However, pioglitazone protected against the PC-induced reduction of the COX4 level and complex I/IV activities through the expression of PINK-1 (Figure 5A–D). In addition, fluorescence-activated cell sorting (FACS) analysis for MitoSOX staining revealed that PC induced ROS generation in MSCs, whereas pioglitazone significantly decreased PC-induced ROS generation with respect to the PC-treated MSCs (Figure 5E). Furthermore, under the PC exposure condition, silencing of PINK-1 in pioglitazone-treated MSCs significantly induced ROS generation compared with treatment of MSC with pioglitazone alone (Figure 5E).

**Figure 6:**
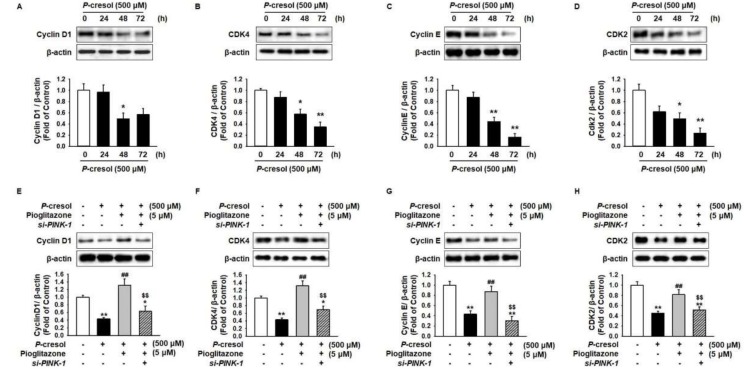
Pioglitazone-treated MSCs increased cell proliferation by regulation of PINK-1. (**A**–**D**) Western blot was used to analyze the expression of cell-cycle-associated proteins cyclin D1, cyclin-dependent kinase 4 (CDK4), cyclin E, and CDK2 in MSCs on exposure to PC (500 μM) for 0, 24, 48, and 72 h. The expression levels were determined by densitometry relative to β-actin. Values represent the mean ± SEM * *p* < 0.05 and ** *p* < 0.01 vs untreated MSCs. (**E**–**H**) After the 72 h PC (500 μM) exposure, Western blot was used to analyze the expression of COX4 in MSCs after the 24 h treatment of *si-PRNP*-transfected MSCs with pioglitazone (5 μM; 24 h). The expression levels were determined by densitometry relative to β-actin. Values represent the mean ± SEM. * *p* < 0.05 and ** *p* < 0.01 vs untreated MSCs, ## *p* < 0.01 vs MSCs exposed to PC, $$ *p* < 0.01 vs MSCs treated with pioglitazone.

Under the PC exposure condition, proliferation significantly decreased via inhibition of cell-cycle-associated proteins, including cyclin D1, cyclin-dependent kinase 4 (CDK4), cyclin E, and CDK2 in PC in a time-dependent manner (Figure 6A–D), whereas pioglitazone ameliorated the PC-induced inhibition of cell-cycle-associated proteins through the expression of PINK-1 (Figure 6E–H).

## 3. Discussion

CKD leads to cardiovascular disease and fibrosis through hyperhomocysteinemia, oxidant stress, dyslipidemia, and elevated inflammation [19]. Exposure to high concentrations of uremic compounds reduces cell proliferation, cell migration, and cell pluripotency in MSCs via mitochondrial dysfunction potential [20,21,22,23]. Recent studies have demonstrated that MSC-based therapy is one of the best candidates for regeneration and healing in CKD [24,25]. MSCs, which are a subcategory of adult stem cells, are widely used for organ regeneration and tissue repair due to their secretion of growth factors and cytokines, capacity for tissue-specific differentiation, and subsequent self-renewal ability [26,27]. This study has shown that pretreatment of MSCs with pioglitazone restored mitochondrial potential, and increased activation of complexes I/IV and mitophagy through up-regulation of PINK-1 under PC exposure conditions. These findings suggest that pretreatment of MSCs with pioglitazone could enhance stem cell therapy for CKD patients with high levels of uremic compounds.

PC is generated by intestinally derived end-products of tyrosine and phenylalanine catabolism. In CKD, PC induces endothelial dysfunction, leading to inhibition of endothelial proliferation and wound repair [28]. Previous studies have revealed that PC suppresses the functionality of human bone marrow MSCs [8]. Our previous studies also indicated that PC led to apoptosis and senescence in MSCs [9,10]. This study revealed that PC induced MSC apoptosis through regulation of pro- and anti-apoptosis-associated proteins, whereas treatment with pioglitazone blocked PC-induced apoptosis. Pioglitazone protected against advanced glycation end product-induced apoptosis in chondrocytes through regulation of mitogen-activated protein kinase and NF-κB [29]. Pioglitazone, as a peroxisome proliferator-activated receptor-gamma (PPAR-γ), reduced apoptosis of bone marrow endothelial progenitor cells via the PI3K–Akt signal axis [30]. Our previous findings have revealed that pioglitazone protects against the uremic toxin, indoxyl-sulfate-induced cellular senescence in MSCs through the PPAR-γ–cellular prion protein signal pathway [31]. These findings suggest that pioglitazone protects against PC-induced apoptosis in MSCs through the regulation of apoptosis-associated proteins.

Mitochondria are one of the pivotal organelles for regulating cellular bioenergetics and apoptosis. Recent studies have found that the protein-bound uremic toxins indoxyl sulfate and PC induced dysfunction of aerobic and anaerobic mitochondrial respiration in vivo and in vitro [32]. Our results indicate that pioglitazone inhibits PC-induced mitochondrial dysfunction through the NF-κB–PINK-1 signal pathway. It is well known that PINK-1 plays an important role in the degradation of damaged mitochondria [33]. Decreasing PINK-1 leads to an accumulation of dysfunctional mitochondria, due to reduced mitophagy in combination with increase in mitochondrial fusion (mitofusion) [34,35]. PINK-1 also maintains mitochondrial function through regulated electron transport chain complex I and IV enzyme activity [33]. Thus, increasing PINK-1 expression may restore mitochondrial turnover pathways that could impact energy production. These findings suggest that pioglitazone may enhance mitochondrial function by activating the NF-κB–PINK-1 axis.

The mitophagy pathway of PINK-1 stabilizes on the outer mitochondrial membrane with collapse of the membrane potential [33]. Recruitment of Parkin ubiquitin proteins can be recognized by P62 and LC3, ultimately leading to mitophagic degradation [36]. However, mitophagy is closely integrated with mitofission. In the CKD condition, mitochondria are poorly enveloped into the autophagosome to eliminate the aberrant mitochondrial fission/fusion dynamics, swollen mitochondria, and dysfunctional mitochondria [16]. Thus, fusion of mitochondria is considered to be harmful in CKD [17,37]. Our data has shown that pioglitazone inhibited PC-induced mitofusion and enhanced mitophagy in MSCs. However, the protective effect of pioglitazone against PC exposure was blocked by knockdown of PINK-1. Silencing of PINK-1 in pioglitazone-pretreated MSCs increased mitofusion and impaired autophagy. These data indicate that pioglitazone repaired PC-induced dysfunction of mitophagy flux through up-regulation of PINK-1.

CKD patients with several mitochondria dysfunctions have more severe pathophysiological conditions and higher levels of ROS, inflammation, and cytokine imbalance [38]. Mitochondrial dysfunction induces the attenuation of mitochondrial membrane potential and accumulation of O_2_^•−^ (superoxide), indicating that mitochondria suppress the production of ATP and consequently have a high Δp (proton motive force) and reduced CoQ (coenzyme Q) pool, and a high NADH/NAD^+^ ratio in the mitochondrial matrix [39]. In particular, the expression of COX4 is essential for respiration of mitochondria for electronic transport chain (ETC) activity [40]. Our findings have shown that PC decreased the level of COX4 and pioglitazone rescued COX4 expression under the PC exposure condition via the expression of PINK-1. In the PINK-1 deficiency model, ETC was impaired by the reduction of complex I and IV activity, resulting in apoptosis [18]. In addition, under the PC exposure condition, pioglitazone increased cell proliferation through the regulation of cell-cycle-associated proteins. These results reveal that pioglitazone enhances the activity of ETC through augmentation of the COX4 level and complex I/IV activity.

**Figure 7:**
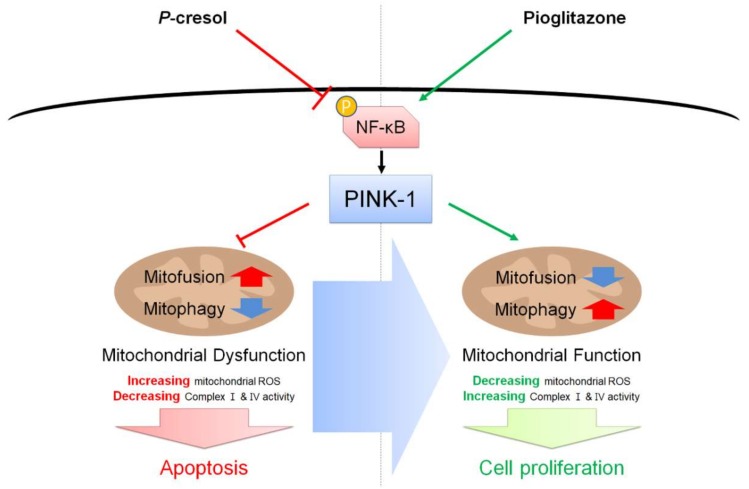
Schematic representation of possible mechanisms by which pioglitazone prevents PC-induced dysfunction of mitochondria in MSCs through the NF-κB–PINK-1 signal pathway. Under the PC exposure condition, the activation of NF-κB and the expression of PINK-1 are decreased, resulting in apoptosis induced by dysfunction of mitochondria. Treatment with pioglitazone activates NF-κB and increases the expression of PINK-1 under the PC exposure condition, leading to the reinstatement of function of mitochondria and the augmentation of MSC proliferation. Red thick arrow means up-regulation, and blue thick arrow means down-regulation.

In conclusion, our findings indicated that pioglitazone reduced cell apoptosis and enhanced cell proliferation in MSCs under exposure to the uremic toxin PC via the NF-κB–PINK-1 signal axis (Figure 7). In particular, we verified that pioglitazone prevents PC-induced dysfunction of mitochondria through inhibition of mitofusion, enhancement of mitophagy, and activation of complexes I and IV. Additionally, we revealed by pretreatment of MSCs with pioglitazone that PINK-1 is a key factor in accumulating dysfunctional mitochondria under high exposure to uremic compounds. These findings suggest that pioglitazone-pretreated MSCs could be an effective candidate for MSC-based therapeutics for patients with CKD, and that understanding of the regulation of NF-κB–PINK-1 may provide important insights regarding mitochondrial potential.

## 4. Materials and Methods

### 4.1. Human MSC Cultures

Human MSCs were obtained from the American Type Culture Collection (ATCC; Manassas, VA, USA). The supplier certified the expression of MSC surface markers CD73 and CD105, and showed that when cultured with specific differentiation media, MSCs differentiate into chondrogenic, adipogenic, and osteogenic forms. Nor-hMSCs and CKD-hMSCs were cultured in alpha-Minimum Essential Medium (α-MEM; Gibco BRL, Gaithersburg, MD, USA) supplemented with 10% (*v*/*v*) fetal bovine serum (FBS; Gibco BRL) and 100 U/mL penicillin/streptomycin (Gibco BRL). MSCs were grown in a humidified 5% CO_2_ incubator at 37 °C.

### 4.2. Flow Cytometry Analysis

To further confirm the characterization of MSCs and PC-treated MSCs, MSCs were subjected to flow cytometry analysis using CD29, CD90, CD105 (MSC-specific markers as positive markers), and CD34 (hematopoietic stem cell marker as a negative marker) antibodies (BD, San Jose, CA, USA) [1]. Flow cytometry was performed using a Cyflow Cube 8 (Partec, Münster, Germany). Data were analyzed using standard FSC Express 5 (De Novo Software, Los Angeles, CA, USA).

### 4.3. Chemical Treatment

MSCs were washed twice with phosphate-buffered saline (PBS), and fresh α-MEM supplemented with 10% FBS was added. To investigate the apoptosis and mitophagy signaling pathway, MSCs were pretreated with pioglitazone (5 µM) at 37 °C for 24 h and then treated with PC (500 µM) for varying time duration (0, 24, 48, and 72 h). To prove NF-κB regulation of PINK-1 expression, MSCs were pretreated with SN-50 (5 µM; NF-κB inhibitor) for 48 h. MSCs were washed twice with PBS, and then these cells were treated with pioglitazone for 48 h.

### 4.4. Inhibition of PINK-1 Expression by RNA Interference

MSCs (2.5 ×  10^5^ cell) were seeded in 6-well plates and were transfected with *si*-RNA in serum-free Opti-MEM (Gibco BRL) using Lipofectamine 2000, following the manufacturer’s instructions (Thermo Fisher, Waltham, MA, USA). At 48 h after transfection, total protein was extracted and the protein expression was determined by Western blot analysis. The *si*-RNA used to target PINK-1 were synthesized by Bioneer (Binoeer, Daejeon, Korea).

### 4.5. Western Blot Analysis

Whole lysates of MSCs (30 μg protein) were separated via 8–12% sodium dodecyl sulfate polyacrylamide gel electrophoresis (SDS-PAGE), and the proteins were transferred to nitrocellulose. After the blots had been washed with TBST (10 mM Tris-HCl (pH 7.6), 150 mM NaCl, 0.05% Tween-20), the membranes were blocked with 5% skim milk for 1 h at room temperature and incubated with the appropriate primary antibodies against B-cell lymphoma 2 (BCL2), BCL-2-associated X protein (BAX), cleaved caspase-3, cleaved Poly(ADP-ribose) polymerase 1 (PARP-1), phospho-NF-κB (p-NF-κB), p-dynamin-1-like protein 1 (DRP1), cyclin D1, cyclin-dependent kinase 4 (CDK4), cyclin E, CDK2, and β-actin (Santa Cruz Biotechnology, Dallas, TX, USA), and PINK-1, mitofusin-1 (MFN1), dynamin-like 120 kDa protein (OPA1), P62, microtubule-associated proteins 1A/1B light chain 3B (LC3B), and cytochrome c oxidase subunit 4 (COX4) (NOVUS, Littleton, CO, USA). The membranes were then washed, and the primary antibodies were detected using goat anti-rabbit IgG or goat anti-mouse IgG conjugated secondary antibodies (Santa Cruz Biotechnology). The bands were detected using enhanced chemiluminescence (Amersham Pharmacia Biotech, Little Chalfont, UK).

### 4.6. Annexin/Propidium Iodide (PI) Assay

After suspended MSCs were washed twice with PBS, these cells were treated in 50 μL assay buffer containing PI and annexin V (Sigma-Aldrich, St. Louis, MO, USA) for 30 min. Apoptosis in MSCs was assessed with a Cyflow Cube 8 (Partec, Munster, Germany) after staining the cells with Annexin V-FITC and PI (De Novo Software, Los Angeles, CA, USA). Data was analyzed using standard FSC Express (De Novo Software, Los Angles, CA, USA).

### 4.7. Mitochondria Fluorescent Staining

MitoTracker (Sigma-Aldrich, St. Louis, MO, USA) was used to observe mitochondria in cultured MSCs. The cells were incubated with MitoTracker (10 nM) for 15 min. After washing with PBS twice, these cells were suspended in 500 μL PBS, and were used to detect the mitochondrion ratio per cell by using FACS (Sysmex, Kobe, Japan). Cell forward scatter levels indicating MitoSOX™ positive cells were analyzed using Flowing Software (De Novo Software, Los Angles, CA, USA).

### 4.8. Autophagy Assay

Autophagy staining (Sigma-Aldrich) was used to observe autophagosomes in cultured MSCs. The cells were incubated with autophagy stain (10 μM) for 30 min. After washing with PBS twice, these cells were fixed in 500 μL 4% PFA (paraformaldehyde) for 1 h. These cells were measured for autophagosomes by using FACS (Sysmex, Kobe, Japan). Autophagosome-positive cells were analyzed by using Flowing Software (De Novo Software, Los Angles, CA, USA).

### 4.9. Measurement of Mitochondrial ROS Generation

To measure the generation of mitochondrial ROS, mitochondrial superoxides of MSCs were indicated using MitoSOX™ (Thermo Fisher). These cells were trypsinized for 5 min and centrifugated at 1200 r/min for 3 min, washed with PBS twice, and then incubated with 10 μM MitoSOX™ solution in PBS at 37 °C for 15 min. Cells were then washed at least twice with PBS. These cells were suspended in 500 μL PBS and MitoSOX™ positive cells were detected using FACS (Sysmex). Cell forward scatter levels indicating MitoSOX™ positive cells were analyzed using Flowing Software (DeNovo Software).

### 4.10. Statistical Analysis

Results are expressed as mean ± standard error of the mean (SEM). All of the experiments were analyzed using one-way analysis of variance (ANOVA). Some comparisons of ≥3 groups were made using Dunnett’s or Tukey’s post hoc test. A *p* value of <0.05 was considered statistically significant.

## 5. Conclusions

In this study, we investigated the increase in cell apoptosis and accumulated dysfunctional mitochondria caused by PC. PINK-1-inducing MSCs pretreated with pioglitazone reduced mitochondrial ROS and enhanced mitochondrial potential through mitophagy. PINK-1 also altered the cell apoptosis pathway to cellular proliferation under PC exposure. The results indicated that regulation of PINK-1 may be the key factor for stem cell therapy in chronic kidney disease.

## Supplementary Materials

The following are available online at http://www.mdpi.com/1422-0067/19/10/2898/s1.
