# Supplementary material for: Pioglitazone Protects Mesenchymal Stem Cells against P-Cresol-Induced Mitochondrial Dysfunction via Up-Regulation of PINK-1
Source: Int J Mol Sci. 2018 Sep 24;19(10):2898. doi: 10.3390/ijms19102898 (PMC6213327; doi:10.3390/ijms19102898)
Supplement: Supplementary file 1 [file ijms-19-02898-s001.pdf]

Supplementary Figure

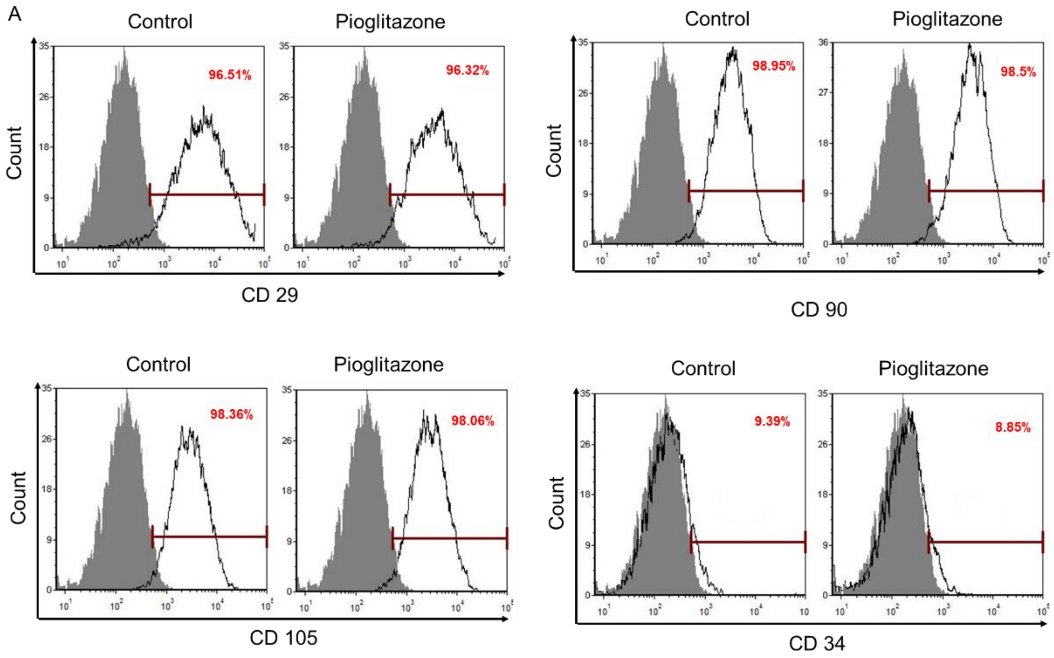

Supplementary Figure S1. Expression of MSC surface markers after treatment of MSCs with Pioglitazone. (A) MSC surface markers, including CD29, CD90, CD105 (MSC specific markers), and CD34 (hematopoietic marker), were analyzed using flow cytometry. Gray lines indicate cell stained with isotype-matched IgG as a negative control. Black lines indicate cell stained with the indicated antibody.
